# Vasoactive inotropic score as a predictor of long-term mortality in patients after off-pump coronary artery bypass grafting

**DOI:** 10.1038/s41598-022-16900-1

**Published:** 2022-07-27

**Authors:** Ji-Hye Kwon, Seung Yeon Yoo, Seonwoo Kim, Hojeong Won, Wooksung Kim, Sukyoung Her, Yu Jeong Bang, Jungchan Park, Jong-Hwan Lee, Hyun Sung Cho, Jeong-Jin Min

**Affiliations:** 1 Department of Anesthesiology and Pain Medicine, Samsung Medical Center, Sungkyunkwan University School of Medicine, 81 Irwon-ro, Gangnam-gu, Seoul, 06351 Korea; 2 Statistics and Data Center, Samsung Medical Center, Seoul, Korea; 3 Department of Surgery, Samsung Medical Center, Sungkyunkwan University School of Medicine, Seoul, Korea

**Keywords:** Cardiology, Risk factors

## Abstract

Increased vasoactive-inotropic score (VIS) is a reliable predictor of mortality and morbidity after cardiac surgery. Here, we retrospectively evaluated the association between VIS and adverse outcomes in adult patients after off-pump coronary artery bypass grafting (OPCAB). We included 2149 patients who underwent OPCAB. The maximal VIS was calculated for the initial 48 postoperative hours using standard formulae. The primary outcome was 1-year death. The composite adverse outcome was death, resuscitation or mechanical support, myocardial infarction, revascularization, new-onset atrial fibrillation, infection requiring antibacterial therapy, acute kidney injury, and stroke. Path-analysis was conducted using lactate and prognostic nutritional index (PNI). VIS was associated with 1-year death (odds ratio [OR] 1.07 [1.04–1.10], *p* < 0.001) and 1-year composite outcome (OR 1.02 [1.0–1.03], *p* = 0.008). In path-analysis, high VIS showed a direct effect on the increased risk of 1-year death and composite outcome. In the pathway using lactate as a mediating variable, VIS showed an indirect effect on the composite outcome but no significant effect on death. Low PNI directly affected the increased risk of 1-year death and composite outcome, and had an indirect effect on both outcomes, even when VIS was used as a mediating variable. In patients undergoing OPCAB, high VIS independently predicted morbidity and 1-year death. Patients with increased lactate levels following high VIS had an increased risk of postoperative complications, although not necessarily resulting in death. However, patients with poor preoperative nutritional status had an increased risk of unfavourable outcomes, including death, implying the importance of preoperative nutritional support.

## Introduction

The vasoactive-inotropic score (VIS) is a reliable predictor of mortality and morbidity after cardiac surgery^1,2^. Although several studies have evaluated the predictive value of VIS in pediatric cardiac surgery^3,4^, there is limited evidence for adult patients undergoing cardiac surgeries, especially those undergoing off-pump coronary artery bypass grafting (OPCAB). Previous studies have shown that a higher VIS predicts unfavourable outcomes in a dose-dependent manner^1–4^.

Although timely initiation of optimal vasopressor and inotrope therapy is essential for patients to maintain adequate tissue perfusion^5^, the use of high-dose vasoactive-inotropic agents can induce impaired tissue perfusion. Blood lactate concentrations have been widely used as markers of altered tissue perfusion, and high blood lactate is associated with major complications after cardiac surgery^6,7^. To date, various independent factors have been proposed, such as VIS and lactate, to predict unfavourable outcomes in coronary artery bypass grafting (CABG), but the causal relationship of variables has not yet been evaluated^8,9^.

Structural equation modelling (SEM) was used to evaluate the causal relationships of significant independent variables. SEM is a multivariate statistical analysis technique that can solve the problem of multicollinearity and identify relationships between explanatory variables, thus developing a more accurate prediction model for the outcome^10^. In the current study, we investigated whether VIS predicted unfavourable outcomes in adult patients after OPCAB. Moreover, we hypothesised that impaired tissue perfusion is the mechanism leading to unfavourable outcomes following high VIS, and consequently, we investigated whether lactate is a mediator between VIS and unfavourable outcomes using SEM. However, although VIS and blood lactate are independent predictors of unfavourable outcomes, they are not modifiable factors; thus, we conducted further analysis to identify any modifiable factors that impact outcomes, such as patients’ prognostic nutritional index (PNI) or any comorbidities.

The aim of this study was to determine independent variables predicting long-term outcomes after OPCAB within 1-year and to investigate causal relationships among independent variables.

## Methods

### Study population

**Figure 1:**
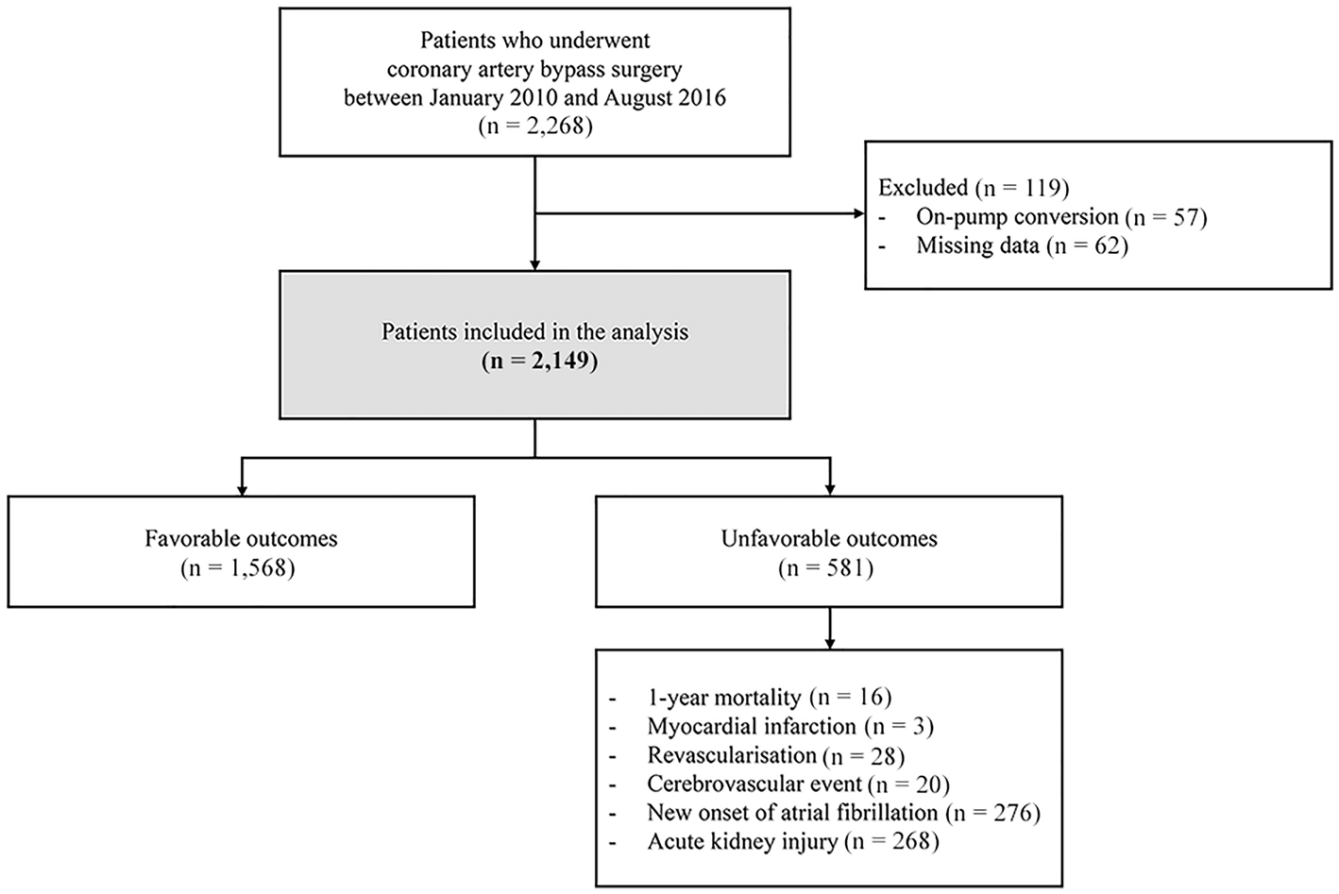
Flowchart of patients.

We performed a single-centre retrospective cohort study including OPCAB patients admitted to the intensive care unit (ICU) at Samsung Medical Center, Seoul, Korea. We screened the records of 2268 patients who underwent OPCAB between January 2010 and August 2016; among whom, 57 patients who underwent on-pump conversion were excluded. Of the remaining 2211 patients, 62 patients were further excluded because of missing data and readmission to the ICU (Fig. 1). This study was approved by the Institutional Review Board of Samsung Medical Center (IRB No. 2020-02-166) and the study was conducted in accordance with the principles of the Declaration of Helsinki and Good Clinical Practice Guidelines. Considering the retrospective nature and minimal risk to participants, the IRB waived the need for individual consent.

### Data collection

From January 2010 to August 2016, 2149 adult patients who underwent OPCAB were enrolled in the analysis. Our institution operates as a paperless hospital with an electronic medical record (EMR) system that archives all patient medication information and laboratory findings. Data in this study were extracted from EMRs and curated using “Clinical Data Warehouse Darwin-C”, an electronic system designed to search and retrieve de-identified medical records. After finalising the patients for inclusion in the study, independent researchers who were blinded to the perioperative medical data organised de-identified data, including baseline characteristics, clinical data, and postoperative outcomes, into a standardised form.

Maximal VIS was calculated (VIS = dopamine dose [mcg/kg/min] + dobutamine [mcg/kg/min] + 100*epinephrine dose [mcg/kg/min] + 10*milrinone dose [mcg/kg/min] + 10,000*vasopressin [units/kg/min] + 100*norepinephrine dose [mcg/kg/min]) using the maximum dosing rates of vasoactive and inotropic medications (mcg/kg/min or IU/kg/min) during the initial 48 postoperative hours after ICU admission. All the data were reviewed and validated by two independent researchers.

Postoperative lactate values and PNI were also collected as mediating variables in SEM. Maximal postoperative lactate was defined as the maximal value of lactate during the initial 48 h after ICU admission. PNI was calculated as 10 × serum albumin (g/dL) + 0.005 × total lymphocyte count (per mm^3^).

All patients were transferred to the specific critical care unit for cardiac surgery patients after OPCAB, and the arterial blood pressure and hemodynamic data measured by the pulmonary artery catheter were continuously monitored. Postoperative patient care was provided at the discretion of the attending ICU physician based on the institutional protocol.

### Study outcomes and definitions

The primary outcome was 1-year death. The secondary outcome was a composite of postoperative complications in the year following surgery, including death, cerebral infarction, cerebral haemorrhage, myocardial infarction (MI), new-onset atrial fibrillation, and postoperative acute kidney injury. Cerebral infarction and cerebral haemorrhage were defined as new neurological deficits with new radiological evidence after cardiac surgery. MI was defined as evidence of coronary thrombus with symptoms or electrocardiographic changes compatible with ischemic aetiology according to the fourth universal definition of MI^11^. Postoperative acute kidney injury (AKI) was defined based on the Kidney Disease Improving Global Outcomes criteria using creatinine level. An absolute increase > 0.3 mg/dl or a relative increase of > 50% from the preoperative baseline level was defined as AKI^12^. Postoperative AKI in our study outcome included both the new onset AKI and aggravating AKI based on the perioperative changes of serum creatinine level. Additionally, subgroup analysis was performed to observe the effects of our study variables on long-term mortality and the composite adverse outcome in separate patient groups with different operation risks. For risk stratification, we used European System for Cardiac Operative Risk Evaluation-II (EuroSCORE-II) system, which is a widely used system for perioperative risk scoring in the cardiac surgery patients^13^. Based on the EuroSCORE-II distribution in our study population, low-risk group was defined as EuroSCORE-II less than 2% and high-risk group was defined as EuroSCORE-II over 2%.

### Statistical analysis

Continuous variables are described as median (interquartile range [IQR]), and categorical variables are expressed as numbers (%). Baseline characteristics were compared between the favourable and unfavourable groups using the chi-square test or Fisher’s exact test for categorical variables and the Wilcoxon rank sum test for continuous variables.

A logistic regression model was used to assess the associations between associated factors and outcomes. A stepwise selection process was performed for multivariable analysis with *p* < 0*.*05 for inclusion of variables, and *p* > 0.10 for removal of variables. The results are described using odds ratios (ORs) with 95% confidence intervals (CIs). The predictability of increased VIS with respect to mortality up to 1 year was assessed by the area under the curve (AUC) of the receiver operating characteristics (ROC) curve.

We used VIS as a continuous variable in the most of our study analysis. However, in the additional analysis to reveal the relationship between VIS and postoperative maximal lactate or the long-term mortality, we used VIS as a categorical variable. We selected VIS cut-off values to define five groups according to the values of an adult population undergoing cardiac surgeries demonstrated in a previous study (five VIS categories: 0–5, > 5–15, > 15–30, > 30–45, and > 45 points)^1^.

In addition to the multivariable logistic regression analysis, we applied path analysis among various SEM models to examine the relationships between patient and perioperative variables and postoperative outcomes. The path analysis model includes independent, dependent (outcome variable), and mediating variables (e.g. lactate or PNI). We chose path analysis models that consisted of three explanatory variables. Because VIS was the most important variable in the risk factor of death, VIS was set as an independent variable, and a composite outcome, or 1-year death was set as the dependent variable. Since postoperative lactate levels indicate tissue perfusion after surgery, lactate was used as the mediating variable in the model. The same analysis was conducted for composite outcome and death. Because there were different results in the indirect effect through lactate between the 1-year death and a composite outcome, we assumed that a patient may have a different prognosis depending on the patient's own underlying nutritional status even if the patient was treated with a similar dosage of vasoactive-inotropic medication or showed a comparable increase in serum lactate. Moreover, in the original logistic regression, PNI was the only variable included in the final model for both 1-year death and composite outcomes; therefore, we added the PNI in SEM and generated an additional path analysis, including the PNI.

All reported *p *values were two-sided, and statistical significance was set at *p* < 0*.*05. Statistical analyses were performed using SAS (version 9.4; SAS Institute, Cary, NC), AMOS 26.0, SPSS 20.0 (IBM Corp., Chicago, IL), or R 3.5.2 (R Development Core Team, Vienna, Austria; http://www.R-project.org/).

## Results

### Patient characteristics

A flowchart of the patients is shown in Fig. 1. Of the 2149 patients who were included in the final analysis, 1565 received vasoactive or inotropic support within 48 h of ICU admission. In these patients who received cardiovascular drug support, VIS ranged from 0.3 to 108. The median VIS was 5 (IQR 0–10) overall, and 7 (IQR 4–12) among patients who received vasoactive or inotropic medication. Distribution of patients (%) and postoperative mortality rate (%) according to the five VIS groups are demonstrated in Supplementary Fig. S1A. Long-term mortality rate after OPCAB increased with higher VIS group (Supplementary Fig. S1A). Overall, 581 (27.1%) patients had one or more postoperative complications within 1 year, 16 patients died, 3 patients experienced myocardial infarction, 28 patients underwent coronary revascularization, 20 patients had cerebrovascular events, 276 patients showed new-onset atrial fibrillation, and 268 patients experienced acute kidney injury (Fig. 1). Compared to patients who had no postoperative complications, patients who had any of the composite outcomes were older and more likely to have a history of stroke and dialysis (Table 1).

**Table 1:** Patient and clinical characteristics according to death and unfavourable outcome.

	All patients (n = 2149)	Unfavourable outcome		*p* value
		No (n = 1568)	Yes (n = 581)	
Male sex	1672 (77.8)	1219 (77.7)	453 (78.0)	0.911
Age (years)	64.0 (57.0–71.0)	63.0 (56.0–70.0)	66.0 (59.0–72.0)	< 0.001
Smoking	569 (26.5)	408 (25.7)	166 (28.6)	0.181
BMI	24.5 (22.8–26.6)	24.5 (22.8–26.5)	24.6 (22.9–26.7)	0.302
**Comorbidities**				
Hypertension	1705 (79.3)	1237 (78.9)	468 (80.55)	0.398
Diabetes	962 (44.8)	689 (43.9)	273 (47.0)	0.207
History of previous MI	187 (8.7)	140 (8.9)	47 (8.1)	0.540
Acute MI	249 (11.6)	173 (11.03)	76 (13.1)	0.188
Previous PCI	378 (17.6)	287 (18.3)	91 (15.7)	0.153
Previous CABG	9 (0.42)	7 (0.45)	2 (0.34)	> 0.999
PAOD	114 (5.3)	75 (4.8)	39 (6.7)	0.076
COPD	28 (1.3)	18 (1.2)	10 (1.7)	0.298
History of stroke	272 (12.7)	178 (11.4)	94 (16.2)	0.003
Chronic kidney disease	122 (5.7)	82 (5.2)	40 (6.9)	0.141
Dialysis	61 (2.8)	37 (2.4)	24 (4.1)	0.028
Cancer	70 (3.3)	46 (2.9)	24 (4.1)	0.165
Heart failure	29 (1.4)	20 (1.3)	9 (1.6)	0.626
Valve disease	10 (0.5)	6 (0.4)	4 (0.7)	0.474
Aortic disease	15 (0.7)	11 (0.7)	4 (0.7)	> 0.999
PTE DVT	2 (0.1)	2 (0.1)	0 (0)	> 0.999
**Medication**				
ACEi	141 (6.6)	97 (6.2)	44 (7.6)	0.249
ARB	549 (25.6)	405 (25.8)	144 (24.8)	0.622
Aspirin	1981 (92.2)	1455 (92.8)	526 (90.5)	0.083
BB	710 (33.0)	499 (31.8)	211 (36.3)	0.049
CCB	668 (31.1)	475 (30.3)	193 (33.2)	0.193
Clopidogrel	1242 (57.8)	885 (56.4)	357 (61.5)	0.037
Statin	1106 (51.5)	793 (50.6)	313 (53.9)	0.174
**Intraoperative parameter**				
Anastomosis number	4 (1–8)	4 (1–8)	4 (1–7)	0.314
OP duration	260 (222–307)	260 (221–305)	262.0 (225.0–313.0)	0.255
RBC transfusion	2.0 (1.0–3.0)	2.0 (1.0–3.0)	2.0 (1.0–3.0)	0.712

Values are presented as n (%) or median (interquartile range).

*BMI* body mass index, *MI* myocardial infarction, *PCI* percutaneous coronary intervention, *CABG* coronary artery bypass grafting, *PAOD* peripheral artery occlusion disease, *COPD* chronic obstructive pulmonary disease, *PTE* pulmonary thromboembolism, *DVT* deep vein thrombosis, *ACEi* angiotensin-converting enzyme inhibitor, *ARB* angiotensin 2 receptor blocker, *BB* beta-blocker, *CCB* calcium channel blocker, *OP* operation, *RBC* red blood cell. Chi-square test or Fisher’s exact test was used for categorical variables and Wilcoxon rank sum test was used for continuous variables.

### Variables associated with the composite outcome

Increased VIS during the immediate postoperative 48 h was independently associated with the occurrence of 1-year composite outcome (adjusted OR 1.02; 95% CI 1.0–1.03; *p* value = 0.008; Table 2). The maximum value of blood lactate within 48 h postoperatively was also independently related to the 1-year composite outcome (adjusted OR 1.21; 95% CI 1.14–1.29; *p* < 0*.*001). In addition, a higher VIS was significantly associated with increased lactate levels in a dose-dependent manner (*p* < 0*.*001; Supplementary Fig. S1B). Preoperative poor PNI was independently associated with the risk of the composite outcome (adjusted OR 0.98; 95% CI 0.96–0.99; *p* = 0*.*011). Multivariable analysis also confirmed a significant association between composite outcome and history of stroke, preoperative infectious state, and preoperative intake of aspirin and beta-blockers (Table 2). The results of subgroup analysis were consistent with the results of entire population (Supplementary Table S1A).

**Table 2:** Logistic regression model for a composite outcome.

	Any unfavourable outcome (n = 581)	No unfavourable outcome (n = 1568)	Univariate analysis		Multivariate analysis	
			Unadjusted OR (95% CI)	*p* value	Adjusted OR (95% CI)	*p* value
VIS	5.0 (2.0–11.0)	5.0 (0.0–9.0)	1.03 (1.02–1.04)	< 0.001	1.02 (1.00–1.03)	0.008
Lactate	2.0 (1.1–2.8)	1.5 (0.8–2.5)	1.24 (1.16–1.31)	< 0.001	1.21 (1.14–1.29)	< 0.001
PNI	51.4 (47.7–55.0)	52.5 (48.9–55.8)	0.96 (0.95–0.98)	< 0.001	0.98 (0.96–0.99)	0.011
**Demographics**						
Male sex	453 (78.0)	1219 (77.7)	0.99 (0.79–1.24)	0.911		
Age (years)	66.0 (59.0–72.0)	63.0 (56.0–70.0)	1.02 (1.01–1.03)	< 0.001	1.01 (1.00–1.02)	0.013
Smoking	166 (28.6)	408 (25.7)	1.16 (0.94–1.43)	0.181		
BMI	24.6 (22.9–26.7)	24.5 (22.8–26.5)	1.01 (0.99–1.05)	0.303		
**Comorbidities**						
Hypertension	468 (80.55)	1237 (78.9)	1.11 (0.87–1.41)	0.397		
Diabetes	273 (47.0)	689 (43.9)	1.13 (0.93–1.37)	0.207		
History of previous MI	47 (8.1)	140 (8.9)	0.90 (0.64–1.27)	0.54		
Acute MI	76 (13.1)	173 (11.03)	1.21 (0.91–1.62)	0.188		
Previous PCI	91 (15.7)	287 (18.3)	0.83 (0.64–1.07)	0.154		
Previous CABG	2 (0.34)	7 (0.45)	0.77 (0.16–3.72)	0.745		
PAOD	39 (6.7)	75 (4.8)	1.43 (0.96–2.14)	0.078		
COPD	10 (1.7)	18 (1.2)	1.51 (0.69–3.29)	0.301		
History of stroke	94 (16.2)	178 (11.4)	1.51 (1.15–1.98)	0.003	1.33 (1.01–1.76)	0.045
Chronic kidney disease	40 (6.9)	82 (5.2)	1.34 (0.91–1.98)	0.142		
Dialysis	24 (4.1)	37 (2.4)	1.78 (1.06–3.01)	0.030		
Cancer	24 (4.1)	46 (2.9)	1.43 (0.86–2.36)	0.167		
Heart failure	9 (1.6)	20 (1.3)	1.22 (0.55–2.69)	0.626		
Valve disease	4 (0.7)	6 (0.4)	1.81 (0.51–6.42)	0.361		
Aortic disease	4 (0.7)	11 (0.7)	0.98 (0.31–3.09)	0.974		
PTE DVT	0 (0)	2 (0.1)	0.54 (0.00–6.63)	0.744		
Infection (CRP pre)	0.1 (0.1–0.5)	0.2 (0.1–0.6)	1.09 (1.04–1.14)	< 0.001	1.06 (1.01–1.1)	0.022
Haemoglobin			0.93 (0.88–0.98)	0.004		
Creatinine			1.01 (0.94–1.08)	0.865		
**Medication**						
ACEi	44 (7.6)	97 (6.2)	1.24 (0.86–1.80)	0.25		
ARB	144 (24.8)	405 (25.8)	0.95 (0.76–1.18)	0.622		
Aspirin	526 (90.5)	1455 (92.8)	0.74 (0.53–1.04)	0.084	0.68 (0.48–0.96)	0.029
BB	211 (36.3)	499 (31.8)	1.22 (1.01–1.49)	0.049	1.25 (1.01–1.53)	0.037
CCB	193 (33.2)	475 (30.3)	1.15 (0.93–1.40)	0.193		
Clopidogrel	357 (61.5)	885 (56.4)	1.23 (1.01–1.49)	0.037		
Statin	313 (53.9)	793 (50.6)	1.14 (0.94–1.38)	0.174		
**Operation**						
Anastomosis number	4 (1–7)	4 (1–8)	0.92 (0.75–1.13)	0.444		
OP duration	262.0 (225.0–313.0)	260 (221–305)	1.00 (1.00–1.00)	0.191		
RBC transfusion	2.0 (1.0–3.0)	2.0 (1.0–3.0)	1.01 (0.95–1.08)	0.653		

*VIS* vasoactive inotropic score, *PNI* prognostic nutritional index, *BMI* body mass index, *MI* myocardial infarction, *PCI* percutaneous cardiac intervention, *CABG* coronary artery bypass graft, *PAOD* peripheral arterial occlusive disease, *COPD* chronic obstructive pulmonary disease, *PTE* pulmonary thromboembolism, *DVT* deep vein thrombosis, *ACEi* angiotensin-converting enzyme inhibitors, *ARB* angiotensin receptor blocker, *BB* beta-blocker, *CCB* calcium channel blocker, *OP* operation, *RBC* red blood cell.

### Predictors of 1-year death

Increased postoperative VIS was independently associated with the risk of postoperative death within 1-year (adjusted OR 1.06; 95% CI 1.03–1.10; *p* value < 0.001; Table 3). Increased postoperative lactate showed a significant association with the risk of postoperative 1-year death in univariable analysis (OR 1.37; 95% CI 1.14–1.66; *p* = 0*.*001) but failed to be included in the final multivariable model. PNI was significantly associated with postoperative 1-year death in both the univariate and multivariate analyses (OR 0.86; 95% CI 0.80–0.93; *p* value < 0.001, and adjusted OR 0.92; 95% CI 0.84–0.99; *p* value = 0.041, respectively). The preoperative need for dialysis was also associated with the risk of 1-year death (adjusted OR 4.95; 95% CI 1.22–20.16; *p* = 0*.*026; Table 3). In the further analysis using ROC analysis to observe the predictability of VIS on death, the cut-off value of VIS for increased risk of 1-year death was 10.5, with an AUC of 0.82 [95% CI 0.72–0.92; *p* < 0*.*001; Supplementary Fig. S2). The results of subgroup analysis were consistent with the results of entire population except for the insignificance of the association between PNI and 1-year death in low-risk patient group, but it might be explained by the increase of type II error induced by decreased number of deaths following subgroup stratification (Supplementary Table S1B).

**Table 3:** Logistic regression model for 1-year death.

	Death (n = 16)	No death (n = 1875)	Univariate analysis		Multivariate analysis	
			Unadjusted OR (95% CI)	*p* value	Adjusted OR (95% CI)	*p* value
VIS	19.0 (8.0–35.0)	5.0 (0–9.8)	1.08 (1.05–1.11)	< 0.001	1.07 (1.04–1.10)	< 0.001
Lactate	2.60 (1.00–4.15)	1.60 (0.90–2.50)	1.37 (1.14–1.66)	0.001		
PNI	46.9 (40.75–52.85)	52.24 (48.66–55.59)	0.86 (0.80–0.93)	< 0.001	0.92 (0.84–0.99)	0.041
**Demographics**						
Male sex	13 (81.3)	1659 (77.8)	0.81 (0.23–2.85)	0.74		
Age (years)	72.50 (62.0–77.5)	64.0 (56.0–71.0)	1.09 (1.02–1.16)	0.007		
Smoking	3 (18.8)	566 (26.5)	0.64 (0.18–2.25)	0.486		
BMI	24.4 (22.5–25.6)	24.6 (22.8–26.6)	0.90 (0.76–1.07)	0.223		
**Comorbidities**						
Hypertension	13 (81.3)	1692 (79.3)	1.13 (0.32–3.98)	0.85		
Diabetes	6 (37.5)	956(44.8)	0.74 (0.27–2.04)	0.559		
History of previous MI	1 (6.3)	186 (8.7)	0.70 (0.09–5.31)	0.728		
Acute MI	5 (31.3)	244 (11.4)	3.52 (1.21–10.2)	0.021		
Previous PCI	0 (0)	378 (17.7)	0.14 (0.00–1.04)	0.173		
Previous CABG	0 (0)	9 (0.4)	6.78 (0.05–57.5)	0.217		
PAOD	2 (12.5)	112 (5.3)	2.58 (0.58–11.5)	0.214		
COPD	1 (6.3)	27 (1.3)	5.20 (0.66–40.8)	0.117		
History of stroke	4 (25)	268 (12.6)	2.32 (0.74–7.24)	0.148		
Chronic kidney disease	5 (31.3)	117 (5.5)	7.83 (2.68–22.9)	< 0.001		
Dialysis	4 (25)	57 (2.7)	12.1 (3.80–38.8)	< 0.001	4.95 (1.22–20.16)	0.026
Cancer	1 (6.3)	69 (3.2)	2.00 (0.26–15.31)	0.507		
Heart failure EF	0 (0)	29 (1.4)	2.16 (0.02–16.67)	0.601		
Valve disease	0 (0)	10 (0.5)	6.13 (0.05–51.30)	0.239		
Aortic disease	0 (0)	15 (0.7)	4.14 (0.03–33.26)	0.345		
PTE DVT	0 (0)	2 (0.1)	25.8 (0.19–334.16)	0.089		
Infection (CRP pre)	1.02 (0.33–3.26)	0.14 (0.05–0.49)	1.15 (1.05–1.25)	0.002		
Haemoglobin	13.1 (11.7–14.3)	13.4 (12.1–14.5)	0.68 (0.53–0.87)	0.002		
Creatinine	0.90 (0.75–1.13)	0.92 (0.79–1.08)	1.15 (1.05–1.25)	0.002		
**Medication**						
ACEi	2 (12.5)	139 (6.5)	2.05 (0.46–9.11)	0.345		
ARB	4 (25)	545 (25.6)	0.97 (0.31–3.02)	0.96		
Aspirin	14 (87.5)	1967 (92.2)	0.59 (0.13–2.62)	0.489		
BB	4 (25)	706 (33.1)	0.67 (0.22–2.10)	0.495		
CCB	5 (31.3)	663 (31.1)	1.01 (0.35–2.91)	0.989		
clopidogrel	10 (62.5)	1232 (57.8)	1.22 (0.44–3.37)	0.703		
Statin	7 (43.8)	1099 (51.5)	0.73 (0.27–1.97)	0.537		
**Operation**						
Anastomosis number	4 (2–8)	4 (1–8)	1.13 (0.58–2.18)	0.725		
OP duration	239.5 (208.5–302.0)	260.0 (222.0–307.0)	0.99 (0.99–1.01)	0.633		
RBC transfusion	4.0 (3.0–4.0)	2.0 (1.0–3.0)	1.49 (1.19–1.87)	0.001		

*VIS* vasoactive inotropic score, *PNI* prognostic nutritional index, *BMI* body mass index, *MI* myocardial infarction, *PCI* percutaneous cardiac intervention, *CABG* coronary artery bypass graft, *PAOD* peripheral arterial occlusive disease, *COPD* chronic obstructive pulmonary disease, *PTE* pulmonary thromboembolism, *DVT* deep vein thrombosis, *ACEi* angiotensin-converting enzyme inhibitors, *ARB* angiotensin receptor blocker, *BB* beta-blocker, *CCB* calcium channel blocker, *OP* operation, *RBC* red blood cell.

### Causal relationship of VIS, lactate, and PNI on outcomes

**Figure 2:**
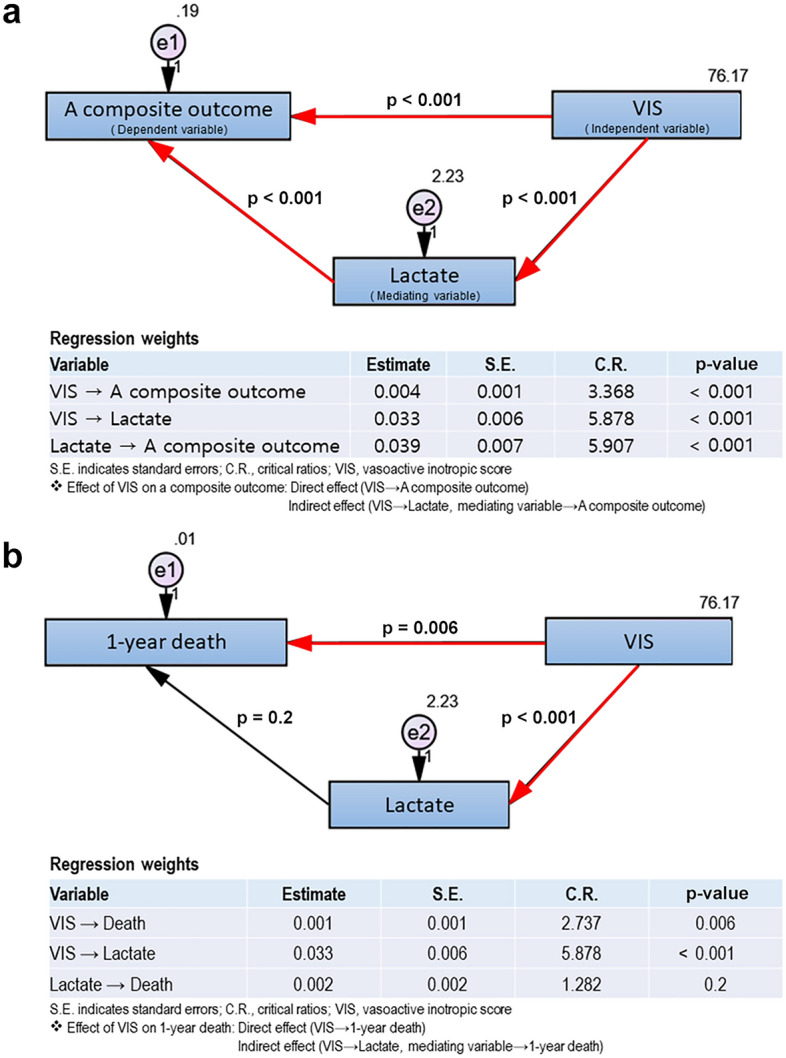
Path analysis using lactate as a mediating variable.

Figure 2 shows the path analysis using postoperative lactate as a mediating variable. High VIS score showed significant effects on the occurrence of the composite outcome directly (*p* < 0*.*001) and indirectly though lactate (*p* < 0*.*001 from VIS to lactate and *p* < 0*.*001 from lactate to composite outcome; Fig. 2a). High VIS also showed a direct significant effect on 1-year death (*p* = 0*.*006) but did not show an indirect effect of lactate on 1-year death (Fig. 2b). In detail, higher VIS had a direct significant effect on the higher postoperative lactate levels (*p* < 0*.*001), but the effect of transiently elevated lactate on the occurrence of 1-year death was insignificant (*p* = 0.2; Fig. 2b).

**Figure 3:**
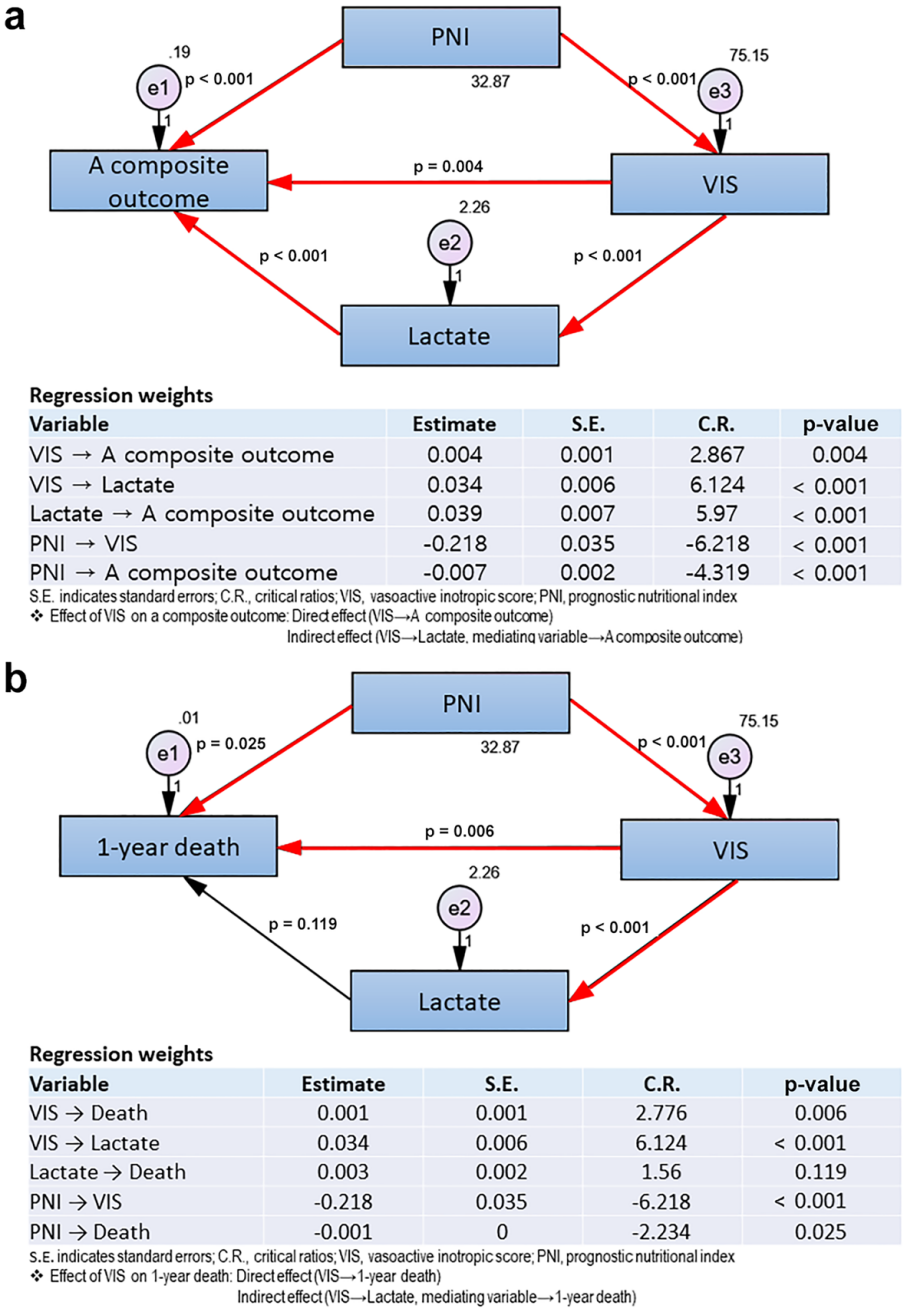
Path analysis using PNI and lactate as a mediating variable.

The PNI was added to the path analysis to determine other factors leading from complications to death (Fig. 3). The PNI showed a significant direct effect on a 1-year composite outcome (*p* < 0*.*001), and also had an indirect effect on the composite outcome when VIS was used as a mediating variable (*p* < 0*.*001 from PNI to VIS and *p* = 0*.*004 from VIS to a composite outcome; Fig. 3a). The PNI also had a significant direct effect on 1-year death (*p* = 0.025; Fig. 3b). In the pathway using VIS as a mediating variable, PNI also showed an indirect effect through VIS on 1-year death (*p* < 0*.*001 from PNI to VIS and *p* = 0.006 from VIS to death; Fig. 3b). There was a consistent trend toward lower PNI values in the death group, although statistical significance was achieved only in patients with a VIS value of ≥ 30 (44 vs. 50, *p* = 0*.*037).

## Discussion

In this retrospective analysis, we found that increased VIS during the immediate postoperative 48 h following OPCAB was significantly associated with long-term morbidity and mortality up to 1-year. In the pathway analysis, increased blood lactate during the immediate postoperative period reflecting temporary impaired tissue perfusion following high VIS may explain the occurrence of various postoperative complications, but not the occurrence of death. However, low PNI was a consistent independent risk factor for both postoperative 1-year death and composite outcome. The pathway using PNI showed that a poor preoperative nutritional state might increase perioperative inotropic requirements, which is consequently related to an increased risk of death and a composite of various complications.

Perioperative VIS is known to be a good predictor of poor clinical outcomes after cardiac surgery in various patient populations^1–4^. In the present study, consistent with previous studies, increased requirement of vasoactive and inotropic agents during the immediate postoperative 48 h following OPCAB also increased the risk of postoperative complications, including death up to 1 year. Moreover, this increased VIS showed a dose–response relationship with postoperative lactate levels. The increase in postoperative lactate may reflect a temporary decrease in systemic organ perfusion, which may be attributed to the prolonged use of various vasopressors, potent inotropic agent support, or the severity of the patient’s illness.

Blood lactate concentration has been widely used as a marker of altered tissue perfusion in critically ill patients^14^. Indeed, previous studies have reported that even minor increases in lactate concentrations are associated with morbidity and higher mortality rates^15^. In the current study, high postoperative blood lactate levels showed a significant association with composite occurrence of various postoperative complications. However, interestingly, the increased blood lactate level was neither a significant risk factor nor could explain the incidence of 1-year death following high VIS. Although blood lactate is a significant predictor of short-term mortality in critically ill patients^6,16–18^, a more complex process may be involved in the occurrence of longer-term mortality after OPCAB, and other important factors may also play a crucial role in long-term death.

Because poor preoperative PNI was consistently associated with the occurrence of both postoperative complications and death in multivariable models, we performed further pathway analysis, including PNI as a mediating variable. Low PNI, which refers to the decreased response of albumin and lymphocytes to acute disease, indicates a low immune-nutritional status, which consequently induces depression in cellular immunity associated with surgical events^19^. The increase in postoperative morbidity and mortality following poor PNI can be explained by the functional alterations in the immune system associated with surgical stress and is related to a shift in the physiological homeostatic balance between pro-inflammatory and regulatory cytokines^19,20^. Because poor nutritional status has many clinical consequences, including decreased quality of life, impaired ambulation, reduced treatment response, and increased treatment-related toxicity, previous studies have reported the significance of PNI on long-term outcomes, including death^21–23^. Accordingly, PNI has been widely used to assess the prognosis of various surgical groups^24–27^. Some studies have suggested that PNI should be considered as a clinical element and indicator of disease severity in patients undergoing CABG^21^. Recently, several studies reported that lower PNI levels were significantly associated with higher mortality and morbidity in patients with cardiovascular disease, including coronary artery disease, undergoing CABG^21,28^. In the current study, PNI was also an independent risk factor for morbidity and mortality.

The current study has strength in that the study population was more homogenous than that included in previous studies^29^, given that all patients underwent off-pump CABG and the surgery was performed by three experienced surgeons. Because patients with coronary artery disease who undergo CABG have a distinct nature compared to other patients with structural heart disease, there is a need to test the predictability of VIS in this population. Given that developing a validated evaluation tool to predict an operative outcome that could be applied evenly to patients in the high-risk vs. low-risk groups is demanding, the results of the present study is worthwhile. The result of subgroup analysis showed that VIS could be useful predictor for post-operative outcomes in both high-risk and low risk patients undergoing off-pump CABG. Insignificant result of the association between PNI and 1-year death might be explained by increase of type II error induced by decreased number of deaths following subgroup stratification. Furthermore, we applied SEM, which has an advantage in that it can solve the problem of multicollinearity, referring to a situation in which two or more explanatory variables in a multiple regression model are highly linearly related^10^. The multivariable regression model showed that VIS, lactate, and PNI were related to the outcome; however, these explanatory variables were correlated. Moreover, the simple regression model used in previous studies cannot explain the cause-and-effect relationship among these variables but can only show a simple relationship.

This study had several limitations. First, as a retrospective study, we could not exclude the possibility of bias from unanalysed (unmeasured or unmeasurable) variables. Although we attempted to include currently known confounders, we cannot rule out the existence of other confounders. Second, this was a single-centre study that evaluated cardiac surgical patients undergoing OPCAB, and, as a result, our results may not be applicable to different surgical populations. Third, the VIS was calculated as the sum of the maximum dosing rate of all administered inotropes or vasopressors during the first 48 h; therefore, VIS cannot be used to evaluate the individual influence of each drug on the outcome.

In conclusion, in patients undergoing OPCAB, high postoperative VIS independently predicted long-term morbidity and mortality up to 1 year after surgery. Patients with increased lactate levels, reflecting temporary tissue hypoperfusion following high postoperative VIS, had an increased risk of postoperative complications, but they did not necessarily lead to longterm death. However, patients with poor preoperative nutritional status had an increased risk of unfavourable outcome occurrences, including death up to 1-year (Supplementary Fig. S3). Our results imply that preoperative nutritional support can change the postoperative outcome including death after OPCAB.

## Supplementary Information

Supplementary Legends.

Supplementary Figure S1.

Supplementary Figure S2.

Supplementary Figure S3.

Supplementary Table S1.

## Data Availability

The datasets generated during and analyzed during the current study are available from the corresponding author on reasonable request.
