# Supplementary material for: Vasoactive inotropic score as a predictor of long-term mortality in patients after off-pump coronary artery bypass grafting
Source: Sci Rep. 2022 Jul 27;12:12863. doi: 10.1038/s41598-022-16900-1 (PMC9329300; doi:10.1038/s41598-022-16900-1)
Supplement: Supplementary file 1 — Supplementary Legends. [file 41598_2022_16900_MOESM1_ESM.docx]

**Supplementary Figure Legends**

**Supplementary Figure 1.**

(A) Patient distribution and mortality according to the VIS groups

(B) Box plot of correlation between VIS and lactate according to the VIS groups

**Supplementary Figure 2.** Receiver operating curves (ROC) of 1-year death on VIS.

**Supplementary Figure 3.** Summary of results
