# Supplementary figures and images for: Vasoactive inotropic score as a predictor of long-term mortality in patients after off-pump coronary artery bypass grafting
Source: Sci Rep. 2022 Jul 27;12:12863. doi: 10.1038/s41598-022-16900-1 (PMC9329300; doi:10.1038/s41598-022-16900-1)

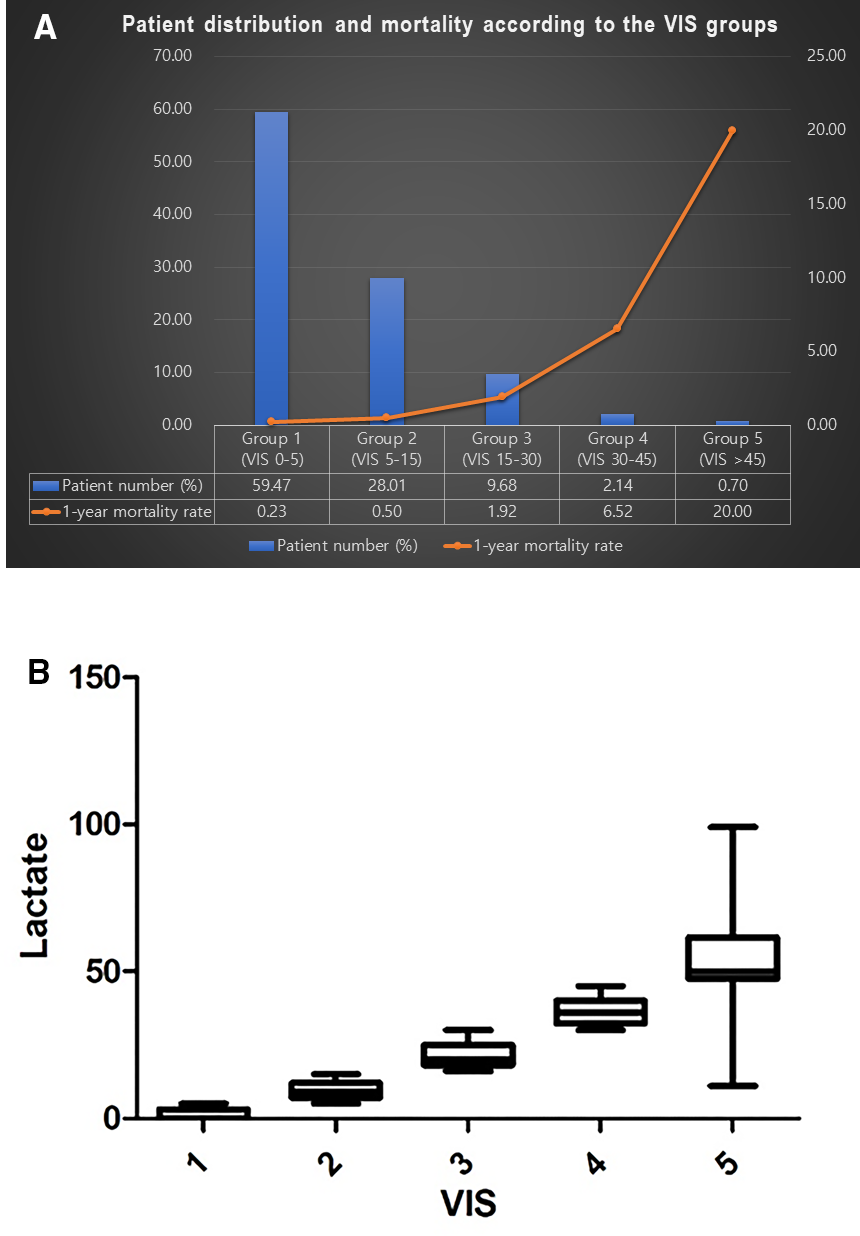

Supplement: Supplementary file 2 — Supplementary Figure S1. [file 41598_2022_16900_MOESM2_ESM.tif]

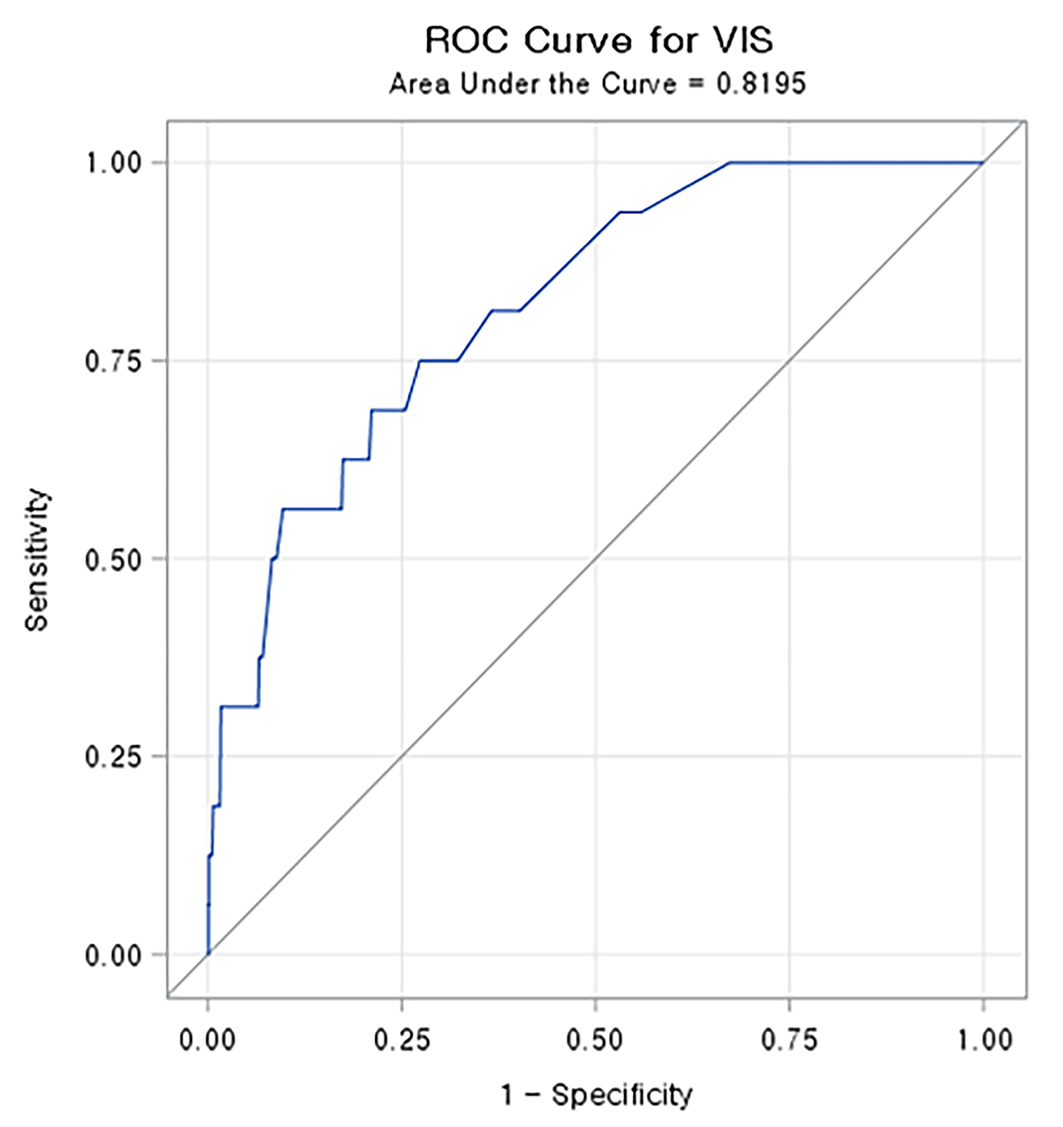

Supplement: Supplementary file 3 — Supplementary Figure S2. [file 41598_2022_16900_MOESM3_ESM.tif]

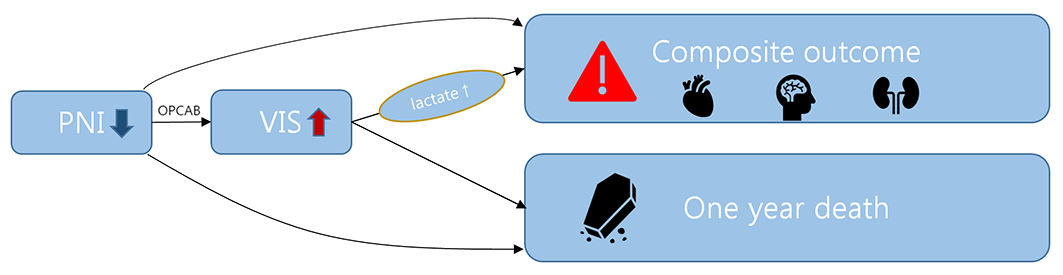

Supplement: Supplementary file 4 — Supplementary Figure S3. [file 41598_2022_16900_MOESM4_ESM.tif]
