# Supplementary material for: Vasoactive inotropic score as a predictor of long-term mortality in patients after off-pump coronary artery bypass grafting
Source: Sci Rep. 2022 Jul 27;12:12863. doi: 10.1038/s41598-022-16900-1 (PMC9329300; doi:10.1038/s41598-022-16900-1)
Supplement: Supplementary file 5 — Supplementary Table S1. [file 41598_2022_16900_MOESM5_ESM.docx]

**Supplementary Table S1. Logistic regression model in low-risk and high-risk subgroups**

| 1. **A composite outcome** | | |  |  |  |  |  |  |  |
| --- | --- | --- | --- | --- | --- | --- | --- | --- | --- |
|  | **Low risk group** | | | |  | **High risk group** | | | |
|  | Univariate analysis |  | Multivariate analysis | |  | Univariate analysis |  | Multivariate analysis | |
|  | Unadjusted OR (95% CI) | p-value | Adjusted OR  (95% CI) | p-value |  | Unadjusted OR (95% CI) | p-value | Adjusted OR  (95% CI) | p-value |
| **VIS** | 1.02 (1.01-1.04) | <0.001 | 1.01 (1.00-1.03) | 0.05 |  | 1.03 (1.01-1.05) | 0.003 | 1.02 (1.00-1.04) | 0.031 |
| **Lactate** | 1.23 (1.14-1.32) | <0.001 | 1.21 (1.12-1.29) | <0.001 |  | 1.25 (1.11-1.42) | <0.001 | 1.22 (1.07-1.39) | 0.003 |
| **PNI** | 0.97 (0.95-0.99) | <0.001 | 0.98 (0.96-0.99) | 0.020 |  | 0.95 (0.91-0.98) | 0.004 | 0.95 (0.91-0.98) | 0.005 |
|  |  |  |  |  |  |  |  |  |  |
| 1. **One-year death** | | |  |  |  |  |  |  |  |
|  | **Low risk group** | | | |  | **High risk group** | | | |
|  | Univariate analysis |  | Multivariate analysis | |  | Univariate analysis |  | Multivariate analysis | |
|  | Unadjusted OR (95% CI) | p-value | Adjusted OR  (95% CI) | p-value |  | Unadjusted OR (95% CI) | p-value | Adjusted OR  (95% CI) | p-value |
| **VIS** | 1.10 (1.05-1.14) | <0.001 | 1.09 (1.05-1.15) | <0.001 |  | 1.05 (1.02-1.09) | 0.003 | 1.05 (1.01-1.08) | 0.005 |
| **Lactate** | 1.19 (0.81-1.74) | 0.385 |  |  |  | 1.46 (1.15-1.87) | 0.002 |  |  |
| **PNI** | 0.66 (0.77-0.96) | 0.007 |  |  |  | 0.91 (0.82-1.01) | 0.076 |  |  |

*VIS* Vasoactive inotropic score, *PNI* Prognostic nutritional index, *OR* Odds ratio, *CI* Confidence interval.
